# Health Differences Among People With Varying Profiles of Awareness of Positive and Negative Age-Related Changes

**DOI:** 10.1093/geroni/igaa057.1553

**Published:** 2020-12-16

**Authors:** Serena Sabatini, Obioha Ukoumunne, Clive Ballard, Manfred Diehl, Hans-Werner Wahl, Allyson Brothers, Linda Clare

**Affiliations:** 1 University of Exeter, Exeter, England, United Kingdom; 2 Colorado State University, Fort Collins, Colorado, United States; 3 University of Heidelberg, Heidelberg, Baden-Wurttemberg, Germany

## Abstract

Higher awareness of negative age-related changes (AARC-losses) is related to poorer mental and physical health whereas higher awareness of positive age-related changes (AARC-gains) is related to better mental health. Associations of health with AARC-gains and losses have been explored separately, but often people experience gains and losses concurrently. Using latent profile analysis, we identified at the cross-sectional level patterns of AARC-gains and losses and explored whether groups with distinct profiles of AARC-gains and losses differed in physical, mental, and cognitive health, and demographic characteristics. Analyses were based on the large-scale PROTECT study conducted in the UK (N= 6,192; mean age= 66.10(SD= 7.04); 76% women). A four-group solution revealed the best model fit (Akaike’s information criterion= 156,061.93; Bayesian information criterion= 156,418.67); 45% of participants perceived many AARC-gains and few losses (Group 1); 24% of participants perceived moderate AARC-gains and few losses (Group 2); 24% of participants perceived many AARC-gains and moderate losses (Group 3); and 7% of participants perceived many AARC-gains and many losses (Group 4). The four groups differed meaningfully in health; Group 1 was the most healthy, followed by Groups 2, 3 and 4. Participants in Group 1 were most likely to perceive their health as excellent, reported the lowest levels of depression and anxiety, and showed the best cognitive performance. On average participants in Group 1 were younger, and more likely to be female, employed, and married, compared to other groups. Considering the co-existence of gains and losses is important when relating awareness of age-related changes to health.

